# Chitinase from a Novel Strain of *Serratia marcescens* JPP1 for Biocontrol of Aflatoxin: Molecular Characterization and Production Optimization Using Response Surface Methodology

**DOI:** 10.1155/2014/482623

**Published:** 2014-04-09

**Authors:** Kai Wang, Pei-sheng Yan, Li-xin Cao

**Affiliations:** School of Marine Science and Technology, Harbin Institute of Technology, Weihai 264209, China

## Abstract

Chitinase is one of the most important mycolytic enzymes with industrial significance, and produced by a number of organisms. A chitinase producing isolate *Serratia marcescens* JPP1 was obtained from peanut hulls in Jiangsu Province, China, and exhibited antagonistic activity against aflatoxins. In this study, we describe the optimization of medium composition with increased production of chitinase for the selected bacteria using statistical methods: Plackett-Burman design was applied to find the key ingredients, and central composite design of response surface methodology was used to optimize the levels of key ingredients for the best yield of chitinase. Maximum chitinase production was predicted to be 23.09 U/mL for a 2.1-fold increase in medium containing 12.70 g/L colloidal chitin, 7.34 g/L glucose, 5.00 g/L peptone, 1.32 g/L (NH_4_)_2_SO_4_, 0.7 g/L K_2_HPO_4_, and 0.5 g/L MgSO_4_ *·*7H_2_O. Polymerase chain reaction (PCR) amplification of the JPP1 chitinase gene was performed and obtained a 1,789 bp nucleotide sequence; its open reading frame encoded a protein of 499 amino acids named as ChiBjp.

## 1. Introduction

Chitin is the second most abundant renewable carbohydrate polymer in nature after cellulose and possibly the most abundant in marine environments [1]. It largely exists in wastes from processing of marine food products [2], with an annual recovery of 1–100 billion metric tonnes as chitinous waste [3]. Most of the chitinous waste is disposed through ocean dumping, incineration, and land filling. The lack of cheap and commercially feasible methods for chitinous processing leads to economic loss, wastage of natural resource, and problem of environmental pollution. Disposal by microbial degradation of chitin offers the best solution to the problem leading to recycling of nutrients in the environment [4, 5].

Chitinases (EC 3.2.1.14) are glycosyl hydrolases which catalyze the first step in chitin digestion. Recently chitinases have received increasing attention because of their wide range of biotechnological applications, especially in agriculture for biocontrol of fungal phytopathogens and harmful insects, because chitin is the essential component of fungal cell wall and cuticle of insects [6–9]. Moreover, the applications of chitinases offer a potential alternative to the chemical fungicides [10, 11]. A wide range of microorganisms could degrade chitin by producing chitinases for nutrition, antagonism, and combating parasites [12–17]. However, to our knowledge there is no report performed on chitinase produced by endophytic* Serratia marcescens* isolated from peanut hulls for biocontrol of aflatoxins production. The ability of* S. marcescens *to produce chitinases is strain dependent and the quantities depended on the nutritional composition of the growth medium and culture condition [18]. Meanwhile the applications of chitinases require large quantities which in turn require optimization of nutritive and physical parameter for the selected isolate.

Several statistical and nonstatistical methods are available for optimization of medium constituents. Plackett-Burman and response surface methodology are the most widely used statistical approaches for reducing the time and expense. Central composite design (CCD) was used to determine levels of various parameters with the interrelation between each parameter evolved simultaneously [19]. Studies on medium optimization for chitinases production are the worthwhile technique for multifactor experiments, because they could detect the true optimum of the factor [20–22]. In addition medium composition greatly influenced the microbial production of extracellular chitinase and its interaction plays an important role in the synthesis of the chitinases [23, 24]. The objective of the present work was to characterize the medium of* S. marcescens* JPP1 for maximum antagonistic effect on aflatoxin in favor of the chitinase production, using statistical designs of Plackett-Burman and central composite design of response surface methodology. The molecular properties of the extracellular chitinase were also determined.

## 2. Materials and Methods

### 2.1. Bacterial Strain

Thestrain used in this study was isolated from the peanut hulls collected from the sampling site in Huaian city, Jiangsu Province, China. Itwas identified as* Serratia marcescens* JPP1 based on its morphological and physiological characteristics and 16S rRNA gene sequence analysis. The nucleotide sequence of 16S rRNA gene was submitted to NCBI GenBank database under the accession number JQ308601 [25].

### 2.2. Media Composition

PGY medium: peanut hulls were dried at 40°C and then ground. The ground peanut hulls were boiled with water for 1 h at the final concentration of 2.5% and then centrifuged at 6,600 g at room temperature for 5 min. The supernatant was supplemented with 2% glucose and 0.5% yeast extract and then autoclaved for 20 min at 121°C, pH in nature.* S. marcescens* JPP1 was maintained on solid PGY medium. The stocks were kept in the refrigerator and subcultured at monthly intervals.

### 2.3. Preparation of Colloidal Chitin

Colloidal chitin was prepared from pure chitin (Sangon Biotech, China) by the method of Roberts and Selitrennikoff [26]. Commercial chitin (40 g) was weighed and taken in a beaker; 500 mL of concentrated hydrochloric acid was added, followed by continuous stirring at 4°C. After stirring for 1 h, the hydrolyzed chitin in the beaker was washed several times with distilled water to remove the acid completely and hence bring the pH into the range of 6-7. Once this pH was obtained, the colloidal chitin was filtered using Whatman filter paper. The filtered colloidal chitin was then collected and stored in the form of a paste at 4°C.

### 2.4. Chitinase Activity Assay

Chitinase activity was tested according to the method of Monreal and Reese detecting N-acetylglucosamine (NAG) as the final product [27]. The reaction mixture for the chitinase assay contained 1 mL of 5% acid swollen chitin, 1 mL of 50 mM acetate buffer (pH 5.0), and 1 mL of enzyme solution. The reaction mixture was incubated at 50°C for 1 h and then the reaction was stopped after boiling for 15 min. The mixture was centrifuged at 5,000 rpm for 20 min and the concentration of released NAG was assayed at 530 nm spectrophotometrically, with colloidal chitin as substrate. One unit of chitinase activity was defined as the amount of enzyme that catalyzed the release of 1 *μ*mol of NAG per hour at 50°C. Data is expressed as mean ± SD of three experiments.

### 2.5. Design of Experiment

The optimization of medium constituents to improve chitinase activity of* S. marcescens* JPP1 was carried out in two stages, Plackett-Burman and response surface methodology. Firstly, eight variables including three best carbon sources, three best nitrogen sources, magnesium sulfate, and potassium hydrogen phosphate anhydrous were selected on the basis of their role in chitinase secretion enhancement. The variables having the most significant effect on chitinase activity were identified using a 2-level Plackett-Burman design.

Secondly, response surface methodology was used to optimize the screened components to enhance chitinase activity using central composite design. A 2^4^ full factorial CCD of RSM was employed to optimize the four most significant factors (glucose, peptone, ammonium sulfate, and chitin) for enhancing chitinase activity. The concentrations of 4 variables were previously investigated for chitinase activity using single-factor experiments (data not shown). In this study, the experimental plan consisted of 31 trials and the independent variables were studied (Table 1). All the experiments were done in triplicate and the average chitinase activity was taken as the dependent variables or responses. The data obtained from CCD on chitinase production were subjected to analysis of variance (ANOVA). Then a second-order polynomial equation was fitted to the data by multiple regression procedure. This resulted in an empirical model that related the response measured in the independent variables to the experiment. The behavior of the system was explained by the following quadratic equation:

(1) $$\begin{array}{c} Y=\beta_{0}+\sum \beta_{i}x_{i}+\sum \beta_{ij}x_{i}x_{j}+\sum \beta_{ii}{x_{i}}^{2}, \end{array}$$

where *Y* is predicted response, *x*_*i*_ and *x*_*j*_ are the input variables, *β*_0_ is the intercept term, *β*_*i*_ is the linear effects, *β*_*ii*_ is the squared effects, and *β*_*ij*_ is the interaction term.

The statistical software package Minitab 16.0 (Minitab Inc., State College, PA) was used to analyze the experimental design. The response obtained was statistically evaluated and the model was built based on the variables with confidence levels more than 95%.

### 2.6. Nucleotide Sequence of Chitinase Gene

Genomic DNA from strain* S. marcescens* JPP1 was extracted using a bacterial genomic DNA FastPrep Extraction Kit (Sangon Biotech, China). The oligonucleotides designed using Primer Premier 5.0 on the basis of the published sequence of chitinase (ChiB) gene [28] were synthesized and used for determination of the chitinase gene sequence. The upstream primer (5′-CCAAGACAGGCGGCAGTAAATAAAA-3′) and downstream primer (5′-AAAAGCGATGTCTACAGCCTGATGG-3′) were used to amplify and sequence the CHI gene.

PCR was performed under the following conditions: 94°C for 5 min, followed by 94°C for 1 min, 58°C for 1 min, and 72°C for 1.5 min for 35 cycles and with a final 10 min extension at 72°C. PCR sequencing was performed at Sangon Biotech, Shanghai. In order to confirm the fidelity of the sequence, two independent PCR products were sequenced in both directions. Sequence comparison was performed in the GenBank database using BLAST through the NCBI server. The protein sequence alignment was performed using CLUSTAL_X program of MEGA 5 package.

## 3. Results and Discussion

Achitinase producing isolate JPP1 was obtained from peanut hulls in Jiangsu Province, China. The JPP1 bacterium was a rod-shaped organism that was gram negative, casein hydrolysis, catalase reactivity, and citrate degradation positive. It was identified as* Serratia marcescens *based on biochemical and genetic characteristics. In preliminary studies the strain exhibited antagonistic activity against mycelia growth and subsequent aflatoxin production. The main mechanism of strain JPP1 for biocontrol against the growth of* A. parasiticus* and aflatoxin production was also determined. The strain JPP1 could produce chitinase to degrade phytopathogenic fungal cell walls [25].

Since the ability of* S. marcescens* to produce chitinases is strain dependent and the medium composition greatly influenced the chitinase synthesis, optimization of culture media for chitinase production was performed. The Plackett-Burman design could provide an efficient way of a large number of variables to identify the most important ones. A total of 8 variables were analyzed with regard to their effects on chitinase production using the Plackett-Burman design. Three best carbon sources (glucose, fructose, and beef extract), three best nitrogen sources (ammonium sulfate, peptone, and ammonium chloride), magnesium sulfate, and dipotassium hydrogen phosphate (K_2_HPO_4_) were selected on the basis of their role in chitinase secretion enhancement. The results revealed that the most significant three factors which were more effective in chitinase production were peptone, glucose, and ammonium sulfate (*P* < 0.01) [29].

Central composite design is a very useful tool for determining the optimal level of significant constituents and their interaction. In this study, CCD was used to determine the optimum level of the four selected significant variables (peptone, glucose, chitin, and ammonium sulfate) for the chitinase production. A total of 31 experiments with different combinations of the four selected variables were performed. The design matrix with the corresponding results of CCD experiments, as well as the experimental results, is presented in Table 1. The *P* values for the model (<0.0001) and for “lack of fit” (0.066) suggested that the obtained experimental data were a good fit with the model. By applying multiple regression analysis on the experimental data, the experimental results of the CCD design were fitted with a second-order polynomial equation for chitinase activity. The response of chitinase production (*Y*) by* S. marcescens* JPP1 can be expressed in terms of the following regression equation:

(2) Y=13.23+0.85A+4.21B+0.33C−1.52D +0.08A∗B−0.14A∗C+0.08A∗D +0.04B∗C−0.18B∗D−0.17C∗D −0.51A2−0.19B2−0.22C2−0.61D2,

where *Y* is chitinase production (response); *A*, glucose; *B*, peptone; *C*, chitin; and *D*, (NH_4_)_2_SO_4_.

The second-order response surface model of (2) was checked by an *F*-test, and the results by the analysis of variance (ANOVA) were given in Table 2. The ANOVA of the quadratic regression model demonstrated that the model was highly significant, due to its high *F* value (*F* = 93.19) and a very low probability value (0). The *F* value shows how well the factors describe the variation in the data about their mean. The greater the *F* value was from unity, the more certain it was that the factors explain adequately the variation in the data about their mean, and the estimated factor effects were real. The *P* values were employed to confirm the significance of each coefficient. Regression coefficients and significance were determined by *P* values which were summarized in Table 3. The fit goodness of the model can be checked by the determination coefficient *R* ^2^ (0.9879), indicating that 98.79% of the variability in the response could be explained by the model. The value of the adjusted *R* ^2^ (0.9773) was also very high to advocate for a high significance of the model.

**Figure 1:**
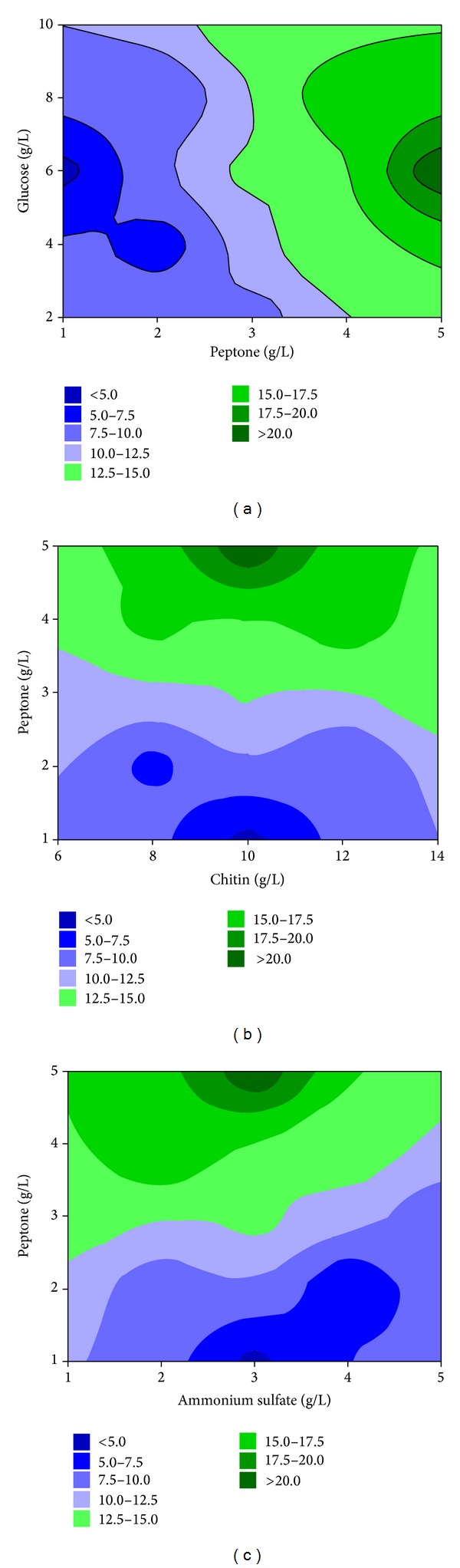
Response surface plot of chitinase production showing the interactive effects of the glucose and peptone concentrations (a), peptone and chitin concentrations (b), and ammonium sulfate and peptone concentrations (c), keeping all other parameters constant.

The 3D response surface was the graphical representation of the regression equation using Minitab 16.0 software. The response surface curves were shown in Figure 1 to help visualize the effects of peptone, glucose, chitin, and ammonium sulfate on chitinase activity, while each figure demonstrated the effect of two factors. From Figure 1, it is evident that an increase in glucose, chitin, and ammonium sulfate concentrations caused enhancement in chitinase secretion, followed by a decrease in its secretion. Figure 1 also shows that maximum chitinase activity was obtained at high concentrations of peptone. In our study, chitinase production was perhaps related to the high concentrations of peptone. The high peptone content would assure the availability of amino acids required for the synthesis of chitinase in general [18].  Figure 1(c) shows the interactive effect of peptone and (NH_4_)_2_SO_4_ concentrations on chitinase production. The chitinase activity increased as (NH_4_)_2_SO_4_ increased from 1 to 1.32 g/L, but further (NH_4_)_2_SO_4_ content showed a declining trend for chitinase production. The medium containing peptone as organic nitrogen source led to the highest chitinase activity, while (NH_4_)_2_SO_4_ was inorganic nitrogen source for chitinase production. Because inorganic nitrogen sources were easily utilized in early bacterial fermentation and organic nitrogen sources could be used in the formation of metabolic enzymes, it preferably includes both organic nitrogen source and inorganic nitrogen source in the medium components.

Because* Serratia* is a member of the Enterobacteriaceae, its direct use as a biocontrol agent would be limited due to its ability to cause disease in humans as an opportunistic pathogen. For this reason, it is important to find cheap and effective culture media that will permit the optimal production of chitinase on a medium scale. The optimized factors for obtaining the highest level of chitinase production were 7.34 g/L glucose, 5.00 g/L peptone, 12.70 g/L chitin, and 1.32 g/L ammonium sulfate. In these conditions, the chitinase production was predicted to be 23.09 U/mL for a 2.1-fold increase.

**Figure 2:**
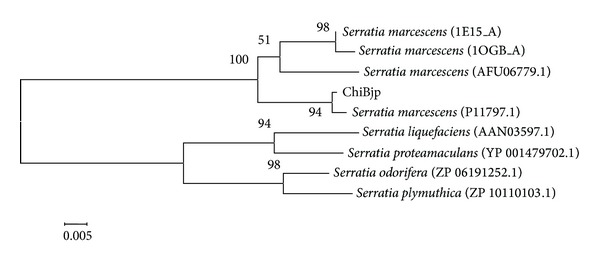
Phylogenetic tree based on the amino acid sequence of ChiB from strain JPP1 and some other related taxa. The numbers on the tree indicate the percentage of bootstrap based on 1,000 replications.

Polymerase chain reaction (PCR) amplification of the chitinase gene was performed and obtained a 1,789 bp nucleotide sequence. The sequence was submitted to NCBI GenBank database under the accession number KJ562867. Similarity search of the nucleotide sequence was performed in the GenBank database using BLASTn. The results showed that the most similar 10 sequences all belonged to* S. marcescens* ChiB gene with the similarities in range of 95–99%. Nucleotide sequencing analysis revealed an open reading frame (ORF) consisting of 1,500 nucleotides with ATG as a start codon and TAA as a stop codon. This ORF encoded a protein of 499 amino acids named as ChiBjp. The deduced protein sequence of ChiBjp was compared with entries in the GenBank database using BLASTp. The N-terminal moiety of ChiBjp showed sequence homology with enzymes classified into family 18 of glycosyl hydrolases such as* S. marcescens* ChiB (WP_016926761.1, sequence identity 99.8%; 3WD0_A, 99.8%) and complex of* S. marcescens* ChiA (1H0G_A, 98.8%). The phylogenetic relationship of ChiBjp with those of representative chitinase from the genus* Serratia* was shown in Figure 2. After similarity alignment and phylogenetic analysis, the chitinase produced by strain JPP1 was determined as ChiB. ChiB is an exochitinase that degrades chitin chains from their nonreducing ends. In addition to the catalytic domain, this enzyme has a small chitin-binding domain that extends the substrate-binding cleft towards the reducing end of the polysaccharide chain [30].

**Figure 3:**
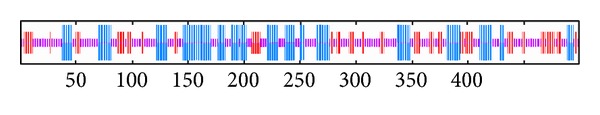
Predicted secondary structure for ChiB from strain JPP1 by GOR4 program (blue: alpha helix; red: extended strand; purple: random coil).

**Figure 4:**
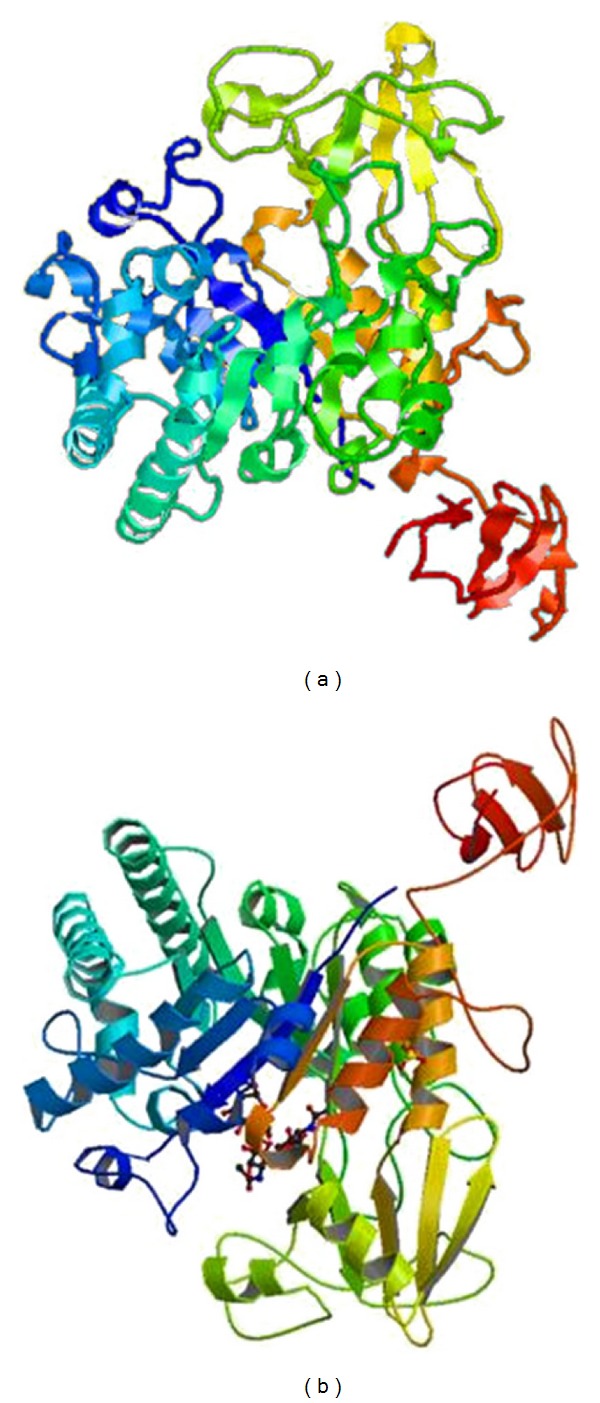
Predicted tertiary structure for ChiB from strain JPP1 by Geno3d program ((a) predicted tertiary structure; (b) template).

Using Protparam program the predicted molecular weight and the isoelectric point of ChiBjp were 55480.3 Da and 5.93, respectively. It was a fat-soluble protein, relatively stable and hydrophilic with formula C_2508_H_3766_N_662_O_738_S_15_. The predicted secondary structure of ChiBjp was 31.66% alpha helix, 20.64% extended strand, and 47.70% random coil using GOR4 program, as shown in Figure 3. The catalytic domains of family 18 chitinases have a (*βα*)_8_ barrel fold. The *β*-strand four of the barrel contains a characteristic DXDXE sequence motif that includes the glutamate residue which protonates that oxygen in the scissile glycosidic bond [31]. Its predicted tertiary structure exhibited similarity of 98.6% with the template by Geno3d program [32], as shown in Figure 4. The template was complex of ChiB (1H0G) from wild type* S. marcescens *with the natural product cyclopentapeptide argadin from* Clonostachys* [33].

## 4. Conclusion

The optimization of medium composition with increased production of chitinase from* Serratia marcescens* JPP1 was carried out using two statistical experimental methods including Plackett-Burman design and central composite design. Maximum chitinase production was predicted to be 23.09 U/mL for a 2.1-fold increase in medium containing 12.70 g/L colloidal chitin, 7.34 g/L glucose, 5.00 g/L peptone, 1.32 g/L (NH_4_)_2_SO_4_, 0.7 g/L K_2_HPO_4_, and 0.5 g/L MgSO_4_ *·*7H_2_O. The results suggested that statistical experimental designs provided an efficient and economical method in optimizing chitinase production for biocontrol of aflatoxins. Similarity alignment and phylogenetic analysis of the nucleotide sequence and deduced amino acid sequence determined that the chitinase produced by strain JPP1 was ChiB. The molecular properties of the chitinase including predicted secondary and tertiary structure were also determined.

## Figures and Tables

**Table 1:** Experimental design and results of CCD of 4 variables in coded and actual units.

Runs	*A*: glucose (g/L)		*B*: peptone (g/L)		*C*: chitin (g/L)		*D*: (NH_4_)_2_SO_4_ (g/L)		Chitinase activity (U/mL)	
	Coded	Conc.	Coded	Conc.	Coded	Conc.	Coded	Conc.	Observed	Predicted
1	−1	4	−1	2	−1	8	−1	2	7.47 ± 0.09	7.56
2	1	8	−1	2	−1	8	−1	2	9.29 ± 0.15	9.24
3	−1	4	1	4	−1	8	−1	2	16.16 ± 0.14	16.11
4	1	8	1	4	−1	8	−1	2	18.18 ± 0.07	18.08
5	−1	4	−1	2	−1	8	1	4	8.79 ± 0.08	8.75
6	1	8	−1	2	−1	8	1	4	9.49 ± 0.10	9.87
7	−1	4	1	4	−1	8	1	4	17.68 ± 0.12	17.45
8	1	8	1	4	−1	8	1	4	18.38 ± 0.03	18.87
9	−1	4	−1	2	1	12	−1	2	5.05 ± 0.06	5.05
10	1	8	−1	2	1	12	−1	2	6.36 ± 0.08	7.03
11	−1	4	1	4	1	12	−1	2	12.83 ± 0.19	12.89
12	1	8	1	4	1	12	−1	2	14.65 ± 0.21	15.17
13	−1	4	−1	2	1	12	1	4	5.04 ± 0.07	5.57
14	1	8	−1	2	1	12	1	4	6.46 ± 0.05	7.00
15	−1	4	1	4	1	12	1	4	13.03 ± 0.17	13.57
16	1	8	1	4	1	12	1	4	14.95 ± 0.14	15.29
17	−2	2	0	3	0	10	0	3	9.5 ± 0.04	9.51
18	2	10	0	3	0	10	0	3	13.84 ± 0.08	12.91
19	0	6	−2	1	0	10	0	3	4.65 ± 0.06	4.05
20	0	6	2	5	0	10	0	3	21.21 ± 0.11	20.89
21	0	6	0	3	0	10	−2	1	11.82 ± 0.08	11.72
22	0	6	0	3	0	10	2	5	13.84 ± 0.03	13.03
23	0	6	0	3	−2	6	0	3	13.64 ± 0.09	13.86
24	0	6	0	3	2	14	0	3	8.89 ± 0.12	7.76
25	0	6	0	3	0	10	0	3	13.43 ± 0.05	13.23
26	0	6	0	3	0	10	0	3	13.13 ± 0.09	13.23
27	0	6	0	3	0	10	0	3	13.74 ± 0.17	13.23
28	0	6	0	3	0	10	0	3	13.64 ± 0.14	13.23
29	0	6	0	3	0	10	0	3	13.03 ± 0.11	13.23
30	0	6	0	3	0	10	0	3	13.04 ± 0.05	13.23
31	0	6	0	3	0	10	0	3	12.63 ± 0.06	13.23

**Table 2:** ANOVA of chitinase activity for the RSM parameters fitted to second-order polynomial equation.

Source of variation	DF	SS	MS	*F* value	*P* value
Model	14	519.02	37.073	93.19	0
*A*	1	17.323	17.323	43.54	0
*B*	1	425.294	425.294	1069.05	0
*C*	1	2.581	2.581	6.49	0.022
*D*	1	55.724	55.724	140.07	0
*A*∗*A*	1	5.105	7.303	18.36	0.001
*B*∗*B*	1	0.362	1.036	2.6	0.126
*C*∗*C*	1	0.658	1.326	3.33	0.087
*D*∗*D*	1	10.522	10.522	26.45	0
*A*∗*B*	1	0.092	0.092	0.23	0.638
*A*∗*C*	1	0.311	0.311	0.78	0.39
*A*∗*D*	1	0.095	0.095	0.24	0.632
*B*∗*C*	1	0.023	0.023	0.06	0.812
*B*∗*D*	1	0.494	0.494	1.24	0.282
*C*∗*D*	1	0.439	0.439	1.1	0.309
Residual	16	6.365	0.398		
Lack of fit	10	5.451	0.545	3.58	0.066
Pure error	6	0.914	0.152		

Total	30	525.385			

*R* ^2^ = 0.9879; DF: degrees of freedom; SS: sum of squares; MS: mean square; Adj *R* ^2^ = 0.9773.

**Table 3:** Test of significance for regression coefficient.

Model term	Coefficient estimate	Standard error	*t* value	*P* value
Intercept	13.2343	0.2384	55.514	0
*A*	0.8496	0.1287	6.599	0
*B*	4.2096	0.1287	32.696	0
*C*	0.3279	0.1287	2.547	0.022
*D*	−1.5237	0.1287	−11.835	0
*A*∗*A*	−0.5053	0.1179	−4.284	0.001
*B*∗*B*	−0.1903	0.1179	−1.614	0.126
*C*∗*C*	−0.2153	0.1179	−1.826	0.087
*D*∗*D*	−0.6066	0.1179	−5.143	0
*A*∗*B*	0.0756	0.1577	0.48	0.638
*A*∗*C*	−0.1394	0.1577	−0.884	0.390
*A*∗*D*	0.0769	0.1577	0.488	0.632
*B*∗*C*	0.0381	0.1577	0.242	0.812
*B*∗*D*	−0.1756	0.1577	−1.114	0.282
*C*∗*D*	−0.1656	0.1577	−1.05	0.309

## References

[1] Bansode VB, Bajekal SS (2006). Characterization of chitinases from microorganisms isolated from Lonar lake. *Indian Journal of Biotechnology*.

[2] Ravi Kumar MNV (2000). A review of chitin and chitosan applications. *Reactive and Functional Polymers*.

[3] Rattanakit N, Plikomol A, Yano S, Wakayama M, Tachiki T (2002). Utilization of shrimp shellfish waste as a substrate for solid-state cultivation of *Aspergillus* sp. S1-13: evaluation of a culture based on chitinase formation which is necessary for chitin-assimilation. *Journal of Bioscience and Bioengineering*.

[4] Gohel V, Maisuria V, Chhatpar HS (2007). Utilization of various chitinous sources for production of mycolytic enzymes by *Pantoea dispersa* in bench-top fermenter. *Enzyme and Microbial Technology*.

[5] Pichyangkura R, Kudan S, Kuttiyawong K, Sukwattanasinitt M, Aiba S-I (2002). Quantitative production of 2-acetamido-2-deoxy-D-glucose from crystalline chitin by bacterial chitinase. *Carbohydrate Research*.

[6] Maisuria VB, Gohel V, Mehta AN, Patel RR, Chhatpar HS (2008). Biological control of *Fusarium* wilt of pigeonpea by *Pantoea dispersa*, a field assessment. *Annals of Microbiology*.

[7] Mathivanan N, Kabilan V, Murugesan K (1998). Purification, characterization, and antifungal activity of chitinase from *Fusarium chlamydosporum*, a mycoparasite to groundnut rust, *Puccinia arachidis*. *Canadian Journal of Microbiology*.

[8] De Pintö AS, Barreto CC, Schrank A, Marilene HV (1997). Purification and characterization of an extracellular chitinase from the entomopathogen *Metarhizium anisopliae*. *Canadian Journal of Microbiology*.

[9] Mendonsa ES, Vartak PH, Rao JV, Deshpande MV (1996). An enzyme from *Myrothecium verrucaria* that degrades insect cuticles for biocontrol of *Aedes aegypti* mosquito. *Biotechnology Letters*.

[10] Huang C-J, Chen C-Y (2008). Synergistic interactions between chitinase ChiCW and fungicides against plant fungal pathogens. *Journal of Microbiology and Biotechnology*.

[11] Bhushan B, Hoondal GS (1999). Effect of fungicides, insecticides and allosamidin on a thermostable chitinase from *Bacillus* sp. BG-11. *World Journal of Microbiology and Biotechnology*.

[12] Ghanem KM, Al-Garni SM, Al-Makishah NH (2010). Statistical optimization of cultural conditions for chitinase production from fish scales waste by *Aspergillus terreus*. *African Journal of Biotechnology*.

[13] Ghanem KM, Al-Fassi FA, Farsi RM (2011). Statistical optimization of cultural conditions for chitinase production from shrimp shellfish waste by *Alternaria alternata*. *African Journal of Microbiology Research*.

[14] Faramarzi MA, Fazeli M, Yazdi MT (2009). Optimization of cultural conditions for production of chitinase by a soil isolate of *Massilia timonae*. *Biotechnology*.

[15] Kern MF, Maraschin SDF, Vom Endt D, Schrank A, Vainstein MH, Pasquali G (2010). Expression of a chitinase gene from *Metarhizium anisopliae* in tobacco plants confers resistance against *Rhizoctonia solani*. *Applied Biochemistry and Biotechnology*.

[16] Patidar P, Agrawal D, Banerjee T, Patil S (2005). Optimisation of process parameters for chitinase production by soil isolates of *Penicillium chrysogenum* under solid substrate fermentation. *Process Biochemistry*.

[17] Yamazaki H, Tanaka A, Kaneko J-I, Ohta A, Horiuchi H (2008). *Aspergillus nidulans* ChiA is a glycosylphosphatidylinositol (GPI)-anchored chitinase specifically localized at polarized growth sites. *Fungal Genetics and Biology*.

[18] Gutiérrez-Román MI, Holguín-Meléndez F, Bello-Mendoza R, Guillén-Navarro K, Dunn MF, Huerta-Palacios G (2012). Production of prodigiosin and chitinases by tropical *Serratia marcescens* strains with potential to control plant pathogens. *World Journal of Microbiology and Biotechnology*.

[19] Lee S-L, Chen W-C (1997). Optimization of medium composition for the production of glucosyltransferase by *Aspergillus niger* with response surface methodology. *Enzyme and Microbial Technology*.

[20] Abdel-Fattah YR, El-Helow ER, Ghanem KM, Lotfy WA (2007). Application of factorial designs for optimization of avicelase production by a thermophilic Geobacillus isolate. *Research Journal of Microbiology*.

[21] Al-Sarrani AQM, El-Naggar MYM (2006). Application of Plackett-Burman factorial design to improve citrinin production in *Monascus ruber* batch cultures. *Botanical Studies*.

[22] El-Naggar MY, El-Aassar SA, Youssef AS, El-Sersy NA, Beltagy EA (2006). Extracellular *β*-mannanase production by the immobilization of the locally isolated *Aspergillus niger*. *International Journal of Agriculture and Biology*.

[23] Al Ahmadi KJ, Yazdi MT, Najafi MF (2008). Optimization of medium and cultivation conditions for chitinase production by the newly isolated: *Aeromonas* sp. *Biotechnology*.

[24] Nawani NN, Kapadnis BP (2005). Optimization of chitinase production using statistics based experimental designs. *Process Biochemistry*.

[25] Wang K, Yan PS, Cao LX, Ding QL, Shao C, Zhao TF (2013). Potential of chitinolytic *Serratia marcescens* strain JPP1 for biological control of *Aspergillus parasiticus* and aflatoxin. *BioMed Research International*.

[26] Roberts WK, Selitrennikoff CP (1988). Plant and bacterial chitinases differ in antifungal activity. *Journal of General Microbiology*.

[27] Monreal J, Reese ET (1969). The chitinase of *Serratia marcescens*. *Canadian Journal of Microbiology*.

[28] Horn SJ, Sørlie M, Vaaje-Kolstad G (2006). Comparative studies of chitinases A, B and C from *Serratia marcescens*. *Biocatalysis and Biotransformation*.

[29] Wang K, Yan PS, Cao LX (2013). Optimization of nutrients for chitinase production by *Serratia marcescens* JPP1 against aflatoxin using statistical experimental design. *Journal of Chemical and Pharmaceutical Research*.

[30] Van Aalten DMF, Synstad B, Brurberg MB (2000). Structure of a two-domain chitotriosidase from *Serratia marcescens* at 1.9-Å resolution. *Proceedings of the National Academy of Sciences of the United States of America*.

[31] Van Aalten DMF, Komander D, Synstad B, Gåseidnes S, Peter MG, Eijsink VGH (2001). Structural insights into the catalytic mechanism of a family 18 exo-chitinase. *Proceedings of the National Academy of Sciences of the United States of America*.

[32] Sahay A, Shakya M (2010). In silico analysis and homology modelling of antioxidant proteins of spinach. *Journal of Proteomics and Bioinformatics*.

[33] Houston DR, Shiomi K, Arai N (2002). High-resolution structures of a chitinase complexed with natural product cyclopentapeptide inhibitors: mimicry of carbohydrate substrate. *Proceedings of the National Academy of Sciences of the United States of America*.

